# The DEAD Box RNA Helicase VBH-1 Is a New Player in the Stress Response in *C. elegans*

**DOI:** 10.1371/journal.pone.0097924

**Published:** 2014-05-20

**Authors:** Daniel Paz-Gómez, Emmanuel Villanueva-Chimal, Rosa E. Navarro

**Affiliations:** Departamento de Biología Celular y Desarrollo, Instituto de Fisiología Celular, Universidad Nacional Autónoma de México, México, Distrito Federal, México; CNRS UMR7622 & University Paris 6 Pierre-et-Marie-Curie, France

## Abstract

For several years, DEAD box RNA helicase Vasa (DDX4) has been used as a bona fide germline marker in different organisms. *C. elegans* VBH-1 is a close homolog of the Vasa protein, which plays an important role in gametogenesis, germ cell survival and embryonic development. Here, we show that VBH-1 protects nematodes from heat shock and oxidative stress. Using the germline-defective mutant *glp-4(bn2)* we found that a potential somatic expression of *vbh-1* might be important for stress survival. We also show that the VBH-1 paralog LAF-1 is important for stress survival, although this protein is not redundant with its counterpart. Furthermore, we observed that the mRNAs of the heat shock proteins *hsp-1* and *sip-1* are downregulated when *vbh-1* or *laf-1* are silenced. Previously, we reported that in *C. elegans*, VBH-1 was primarily expressed in P granules of germ cells and in the cytoplasm of all blastomeres. Here we show that during stress, VBH-1 co-localizes with CGH-1 in large aggregates in the gonad core and oocytes; however, VBH-1 aggregates do not overlap with CGH-1 foci in early embryos under the same conditions. These data demonstrate that, in addition to the previously described role for this protein in the germline, VBH-1 plays an important role during the stress response in *C. elegans* through the potential direct or indirect regulation of stress response mRNAs.

## Introduction

VBH-1 is a member of the broadly conserved family of DEAD box RNA helicases that regulate almost every step of RNA metabolism [1]. These proteins use ATP to rearrange the secondary structure of RNA and ribonucleoprotein (RNP) complexes [2]. In *C. elegans*, VBH-1 is a germline-enriched protein important for fertility and embryonic development [3]. Specifically, VBH-1 promotes the sperm-to-oocyte switch in *C. elegans* and might also be important in apoptosis during oogenesis and under stress [3]–[5]. Furthermore, in the related nematode *Caenorhabditis remanei*, VBH-1 participates in spermatogenesis [5]

*laf-1*, the paralog of *vbh-1*, is more ubiquitously expressed than *vbh-1* in *C. elegans* [6]. This protein plays a role in embryonic development, and together with VBH-1, LAF-1 promotes spermatogenesis [6]. The closest homologs to VBH-1 and LAF-1 in *D. melanogaster* are the RNA helicases Vasa and Belle, which are also important for fertility in this organism [3], [7]–[10]. Vasa promotes mitotic chromosome segregation in the germline of *D. melanogaster* [11] and is required for embryonic patterning and female fertility, as this protein regulates germ cell specification, proliferation, and maintenance [7], [9], [12], [13]. Although Vasa is expressed in the *D. melanogaster* testes, this protein does not play a role in spermatogenesis [13]. Vasa binds to *gurken* and *mei-P26* mRNAs in the germline of *D. melanogaster* and promotes protein translation through the recruitment of the translation initiation factor eIF5B [14]–[16].

Vasa and its orthologs in other species, collectively known as DDX4, are widely used as germline markers in organisms from hydra to humans [17]. However, the expression of DDX4 proteins has recently been detected in somatic tissues, such as human tumors [18], somatic blastomeres in sea urchin [19], and neoblast stem cells in the flatworms *Macrostomum lignano* and *Dugesia japonica* [20]–[22]. The role of DDX4 in somatic cells is not yet clear but these proteins might regulate mitotic cell cycle progression [11], [23].

Belle is more ubiquitously expressed than Vasa and is required for both female and male fertility [8]. Similar to Vasa, Belle promotes mitotic chromosome segregation but in somatic cells [24]. Belle binds to the ecdysone-induced transcription factor *E74A* mRNA and positively regulates its translation [25]. The human homolog of Belle, DDX3, plays a role in tumorigenesis, regulating cell cycle control and apoptosis in somatic cells [26]–[28]. DED1, the homolog of VBH-1 and DDX3 in *Saccharomyces cerevisiae* and *Schizosaccharomyces pombe*, is involved in the response to the depletion of nitrogen or glucose, heat shock, and low temperature potentially through the replacement of the canonical translation initiation factor eIF4A in the unfolding of mRNAs [29]–[31].

In the present study, we observed that *vbh-1(RNAi)* animals are more sensitive to heat shock and oxidative stress than control nematodes, suggesting that VBH-1 plays an important role in the stress response in *C. elegans*. Using the germline-defective mutant *glp-4(bn2)*, we observed that a putative somatic expression of *vbh-1* might be important for this stress response. In addition, we observed that during stress, VBH-1 aggregates into granules in the gonad and early embryos. Moreover, we demonstrated that the mRNAs of the heat shock proteins HSP-1 and SIP-1 are downregulated when *vbh-1* is silenced.

## Results

### VBH-1 is required for stress survival

**Figure 1:**
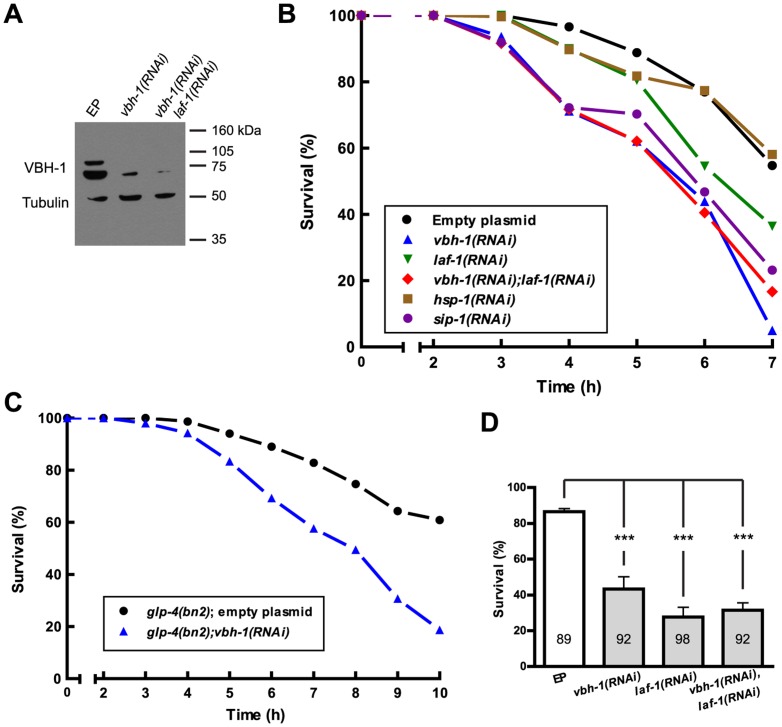
VBH-1 is required for stress survival. (A) Western blot analysis using whole animal protein extracts from 100 hermaphrodites of control (EP, empty plasmid), *vbh-1(RNAi)* and *vbh-1(RNAi);laf-1(RNAi)* animals grown at 25°C. The blot was probed with rabbit anti-VBH-1 and mouse anti-tubulin antibodies as a loading control. (B) Survival curve at 36°C for synchronized hermaphrodites in the indicated background. The data from different experiments were obtained and the percentage of the total was graphed. See Table S1. (C) Survival curve at 36°C for synchronized *glp-4(bn2)* hermaphrodites in the indicated background. The data from three different experiments were obtained, and the percentage of the total was graphed. (D) Survival rate for synchronized hermaphrodites in the indicated background after a 20 min exposure to NGM medium containing hydrogen peroxide. The average from three different experiments was graphed. The bars indicate SEM, *** means statistically significant P<0.001, and the numbers inside each bar indicate the number of animals observed.

**Figure 2:**
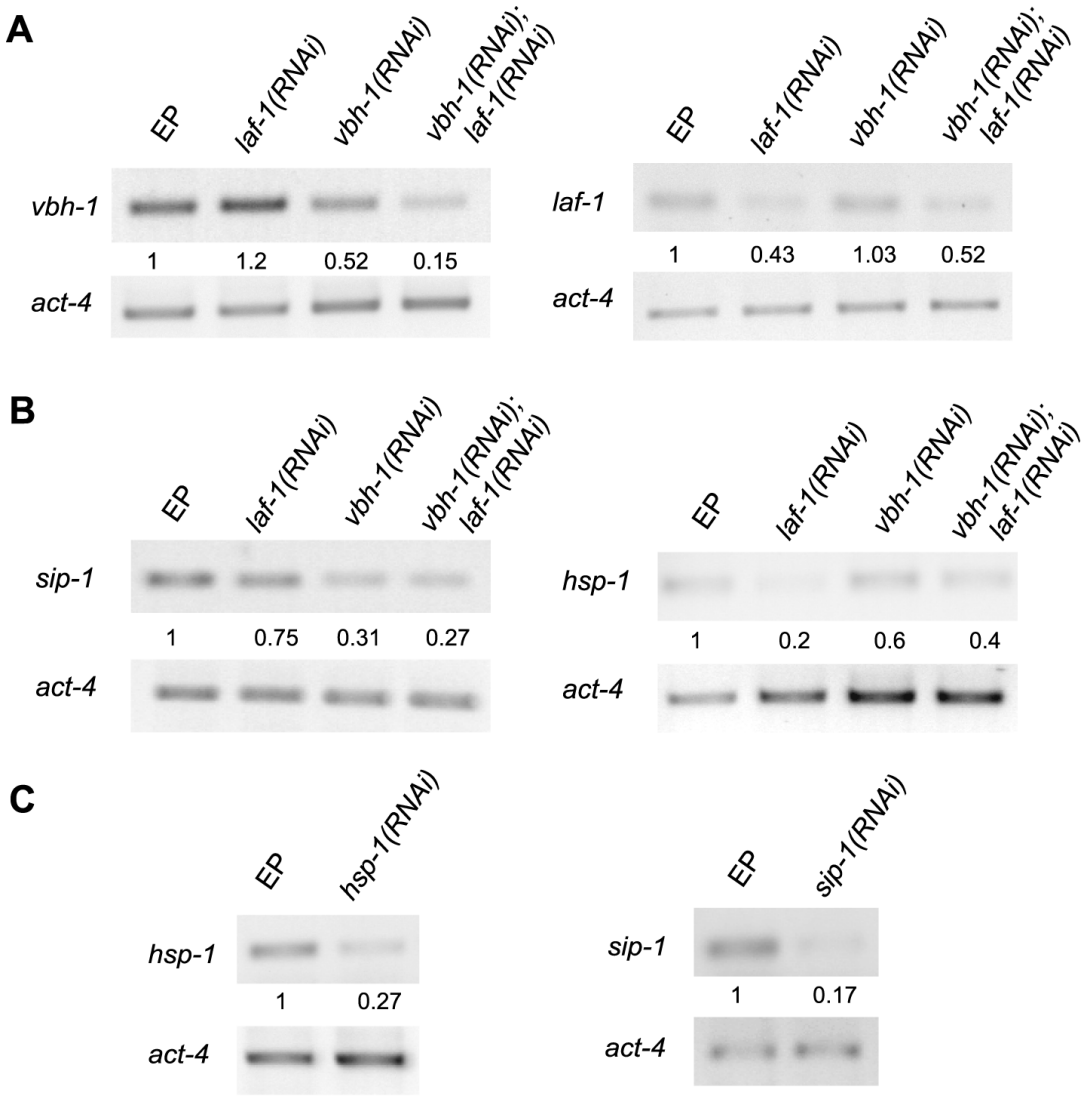
*sip-1* and *hsp-1* mRNAs are downregulated in *vbh-1(RNAi)* and *laf-1(RNAi)* animals. Semi-quantitative RT-PCR analysis using cDNA synthesized from RNA extracts obtained from one-day-old control (EP, empty plasmid), *laf-1(RNAi)*, *vbh-1(RNAi)*, *vbh-1(RNAi);laf-1(RNAi)*, *hsp-1(RNAi)*, and *sip-1(RNAi)* animals. Specific primers for *vbh-1*, *laf-1*, *hsp-1*, *sip-1*, and *act-4* were used. *act-4* was used as a loading control. (A) The efficiency of *vbh-1* and *laf-1 silencing* and (B) downregulation of *sip-1* and *hsp-1* mRNAs in the indicated backgrounds. (C) The efficiency of *hsp-1* and *sip-1* silencing. Densitometric analysis was performed normalizing each band with its corresponding *act-4* band using ImageJ software.

We studied VBH-1 function through RNAi feeding because *vbh-1* null mutants are homozygous lethal (http://www.wormbase.org, release WS236, May 8, 2013). As a negative control for RNAi studies, we used the empty pPD129.36 plasmid (EP) as previously recommended [32]. To evaluate the efficiency of the RNAi, we compared the relative abundance of VBH-1 in protein extracts of control EP and *vbh-1(RNAi)* animals through Western blotting analysis (Figure 1A). An anti-VBH-1 antibody detects several band around 70 kDa through Western blotting, reflecting posttranslational modifications [3]. In the present study, we observed two bands in the whole-protein extracts of control animals (Figure 1A). In the extracts from *vbh-1(RNAi)* animals, the higher molecular weight band was not detected, while the lower molecular weight band was less abundant than in control animals, indicating that *vbh-1* was silenced (Figure 1A). This result was further confirmed through semi-quantitative RT-PCR of control and *vbh-1(RNAi)* animals using *vbh-1* specific oligonucleotides, showing a reduction in the relative abundance of *vbh-1* mRNA (Figure 2A).

To examine the role of VBH-1 in heat shock we used a modified protocol from [33]. One-day-old *vbh-1(RNAi)* and control adult animals were incubated at 36°C, and the mortality of these animals was analyzed every hour. During the first three hours of incubation, no significant difference was observed between *vbh-1(RNAi)* and control animals (Figure 1B and Table S1). After 4 h at 36°C, there were ∼25% less *vbh-1(RNAi)* animals than control animals, and this difference gradually increased with incubation time (Figure 1B and Table S1). After 7 h, only 5% *vbh-1(RNAi)* animals were alive compared with half of the control animals (Figure 1B and Table S1). These data suggest that VBH-1 is important for survival against heat shock in *C. elegans*.

VBH-1 and its paralog LAF-1 are partially redundant in germline sex determination [6]; therefore we examined the role of LAF-1 during heat shock using RNAi. Due to the lack of a specific antibody for LAF-1, the efficiency of the RNAi was examined using semi-quantitative RT-PCR analysis to compare the *laf-1* mRNA levels in control and *laf-1(RNAi)* animals. *laf-1* mRNA was less abundant in *laf-1(RNAi)* animals than in EP animals, suggesting that *laf-1* expression was silenced (Figure 2A). Because of the high degree of homology between *vbh-1* and *laf-1*, it was possible to achieve the unspecific silencing of *laf-1* using *vbh-1* RNAi and vice versa; therefore, we examined this possibility using RT-PCR. We did not observe cross silencing, as the relative amount of *vbh-1* and *laf-1* mRNA was unaffected when silencing *laf-1* and *vbh-1*, respectively (Figure 2A). The sensitivity of *laf-1(RNAi)* animals to heat shock was statistically significant after 6 h of incubation, and ∼20% less animals were alive compared with the control animals. After 7 h of heat shock, only 36% of the animals were alive (Figure 1B and Table S1). These data suggest that LAF-1 is also important for the survival of animals to heat shock.

To determine whether VBH-1 and LAF-1 act redundantly, we silenced both genes using RNAi and tested the roles of these proteins during heat shock. We confirmed the efficiency of this double silencing through Western blotting for *vbh-1* (Figure 1A) and through RT-PCR analysis for *vbh-1* and *laf-1* (Figure 2A). As shown in Figures 1A and 2A, the silencing of both *vbh-1* and *laf-1* using RNAi was efficient. We observed that the silencing of *vbh-1* was more efficient in *vbh-1(RNAi); laf-1(RNAi)* animals than in single *vbh-1(RNAi)* animals (Figures 1A and 2A). This observation potentially reflects the fact that the germline of *vbh-1(RNAi);laf-1(RNAi)* hermaphrodites is more affected than that in *vbh-1(RNAi)* animals; therefore, less *vbh-1* accumulation is observed in these animals. Up to 6 h, *vbh-1(RNAi);laf-1(RNAi)* animals exhibited survival rates similar to *vbh-1(RNAi)* animals at 36°C, but after 7 h at 36°C 17% of the animals remained alive, which was more than the *vbh-1(RNAi)* animals (5% alive) and less than the *laf-1(RNAi)* animals (36% alive, Figure 1B and Table S1). These data indicate that VBH-1 and its paralog LAF-1 are both required during heat shock and, unlike in spermatogenesis, these proteins do not act redundantly.

To determine whether VBH-1 and LAF-1 participate in other types of stress, we exposed RNAi-treated animals to oxidative stress. Control, *vbh-1(RNAi), laf-1(RNAi),* and double *vbh-1(RNAi); laf-1 (RNAi)* one-day-old adult animals were placed on NGM plates with 20 µl of 15% hydrogen peroxide on top and the mortality of the animals was subsequently scored after 20 min. *vbh-1(RNAi)* animals were more sensitive to oxidative stress than control animals, as only 43% *vbh-1(RNAi)* animals were alive compared with 86% control animals (Figure 1D). *laf-1(RNAi)* and *vbh-1(RNAi);laf-1(RNAi)* animals were also more sensitive to oxidative stress than control animals, showing survival rates of 27% and 31% after treatment with hydrogen peroxide (Figure 1D). These data suggest that both VBH-1 and LAF-1 are required but not redundant in the oxidative stress response.

### VBH-1 appears to be expressed in the soma which may contribute to stress protection

We have previously reported that *vbh-1* is germline enriched [3]. Although we did not detect VBH-1 in the somatic cells via immunostaining, the low levels of mRNA detected by Northern blotting suggested the possibility of low levels of protein expression [3]. To determine whether VBH-1 protective function comes from soma, germline, or both, we silenced *vbh-1* in *glp-4(bn2)* animals. The *glp-4* gene product is required for germline proliferation and, when raised at restrictive temperature, *glp-4(bn2)* hermaphrodites are severely depleted of germ cells [34]. If the expression of *vbh-1* in the germline is responsible for the effect of this protein in survival during stress, then *vbh-1* silencing in the germline-deficient mutant *glp-4(bn2)* would not impact survival under these conditions.

We observed that over 80% control *glp-4(bn2)* animals were alive after 7 h at 36°C, compared to only 54% in control wild type animals (Figure 1B and C, Table S1). This result suggests that *glp-4(bn2)* animals are more resistant to heat shock than wild type animals. Despite this, we found that *glp-4(bn2);vbh-1(RNAi*) animals were more sensitive to heat shock than *glp-4(bn2)* control animals (Figure 1C and Table S1), as only ∼19% *glp-4(bn2);vbh-1(RNAi)* animals were alive after 10 h at 36°C compared with 61% control animals. These observations suggest that *vbh-1* expression in somatic tissues could contribute to the stress response. However, given that *glp-4(bn2)* mutants are not completely depleted from the germline, it is also possible that the low *vbh-1* expression in the remaining cells might contribute to survival during heat shock.

To analyze the probable somatic expression of *vbh-1*, we made a VBH-1 transgene under its own regulatory regions fused to the GFP reporter. To generate this transgene, we cloned in tandem a promoter region (608 bp), defined as the intergenic sequence upstream of *vbh-1*, the gfp coding gene without stop codon, and the genomic region from ATG to the end of the longest 3′UTR reported (2228 bp). Transgenic animals were generated using Mos1-mediated single copy insertion (MosSCI) [35], [36]. We obtained two independent transgenic lines expressing the transgene *Pvbh-1::GFP::vbh-1::vbh-1 3*′*utr*: 1) *xmSi05,* with high expression in both soma and germline cells and 2) *xmSi06*, which is partially silenced in the germline. The silencing of single-copy transgenes in the germline is a known problem in *C. elegans* that has been shown to require the piRNA pathway [37].

**Figure 3:**
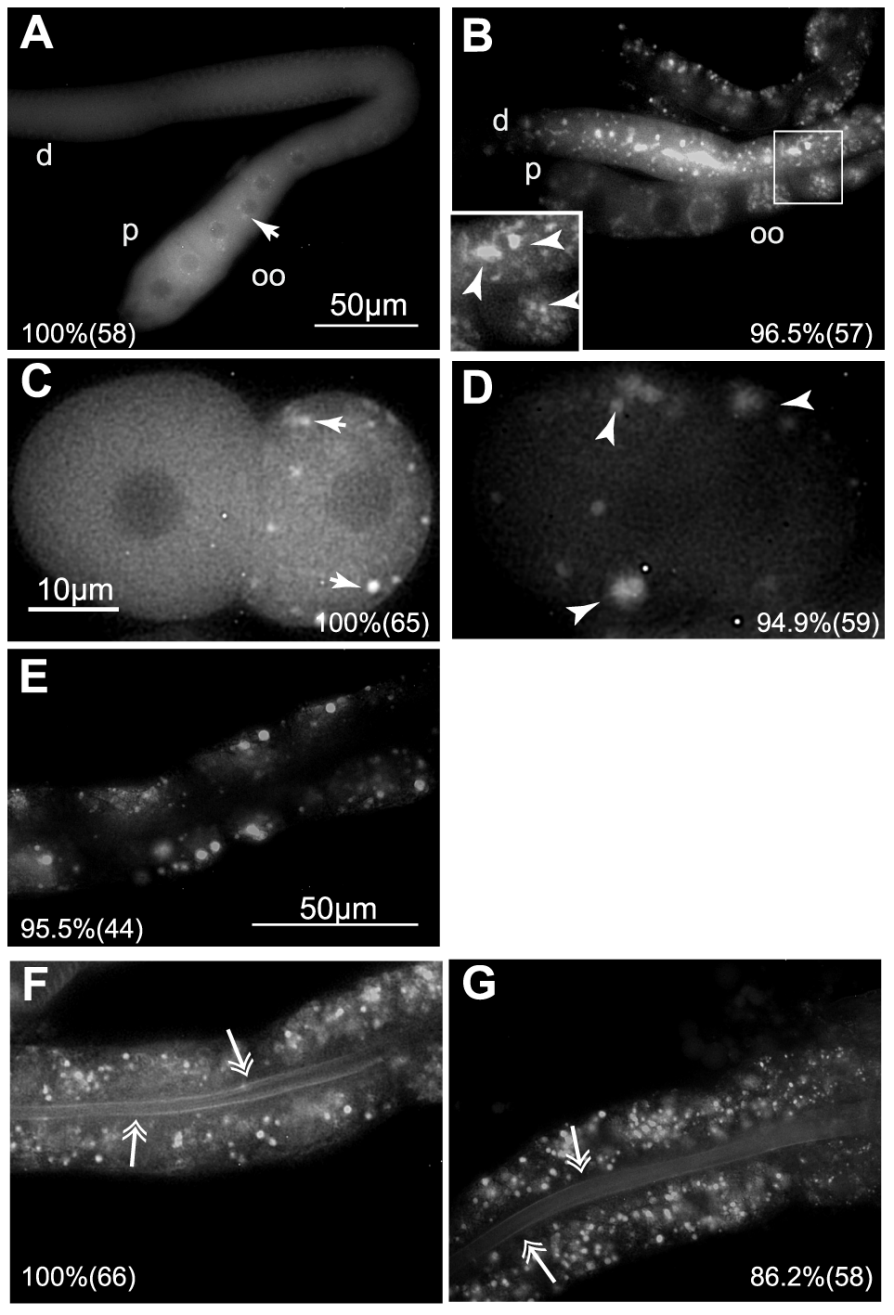
A *GFP::vbh-1* transgene is expressed in the intestine of *C. elegans*. Control (20°C) and heat shocked (30°C for 4 h) animals expressing the transgene *Pvbh-1::GFP::vbh-1::vbh-1 3*′*utr* were dissected and observed under an epifluorescence microscope. Localization of GFP::VBH-1 in (A) the gonads of control animals, (B) gonads after heat shock, (C) 2-cell embryos of control animals, (D) 2-cell embryos of heat shocked animals, and (E–G) guts of animals expressing the transgene *Pvbh-1::GFP::vbh-1::vbh-1 3’utr* (E) after *vbh-1(RNAi),* (F) grown at 20°C, and (G) after heat shock. d =  distal, p =  proximal, oo =  oocytes, the arrows indicate P granules, the arrowheads indicate embryonic foci formed after heat shock, the double arrows indicate the apical pole of intestinal cells. In the lower corner of each panel is indicated the percentage where the depicted phenotype was observed and the number of observed samples in parentheses.

**Figure 4:**
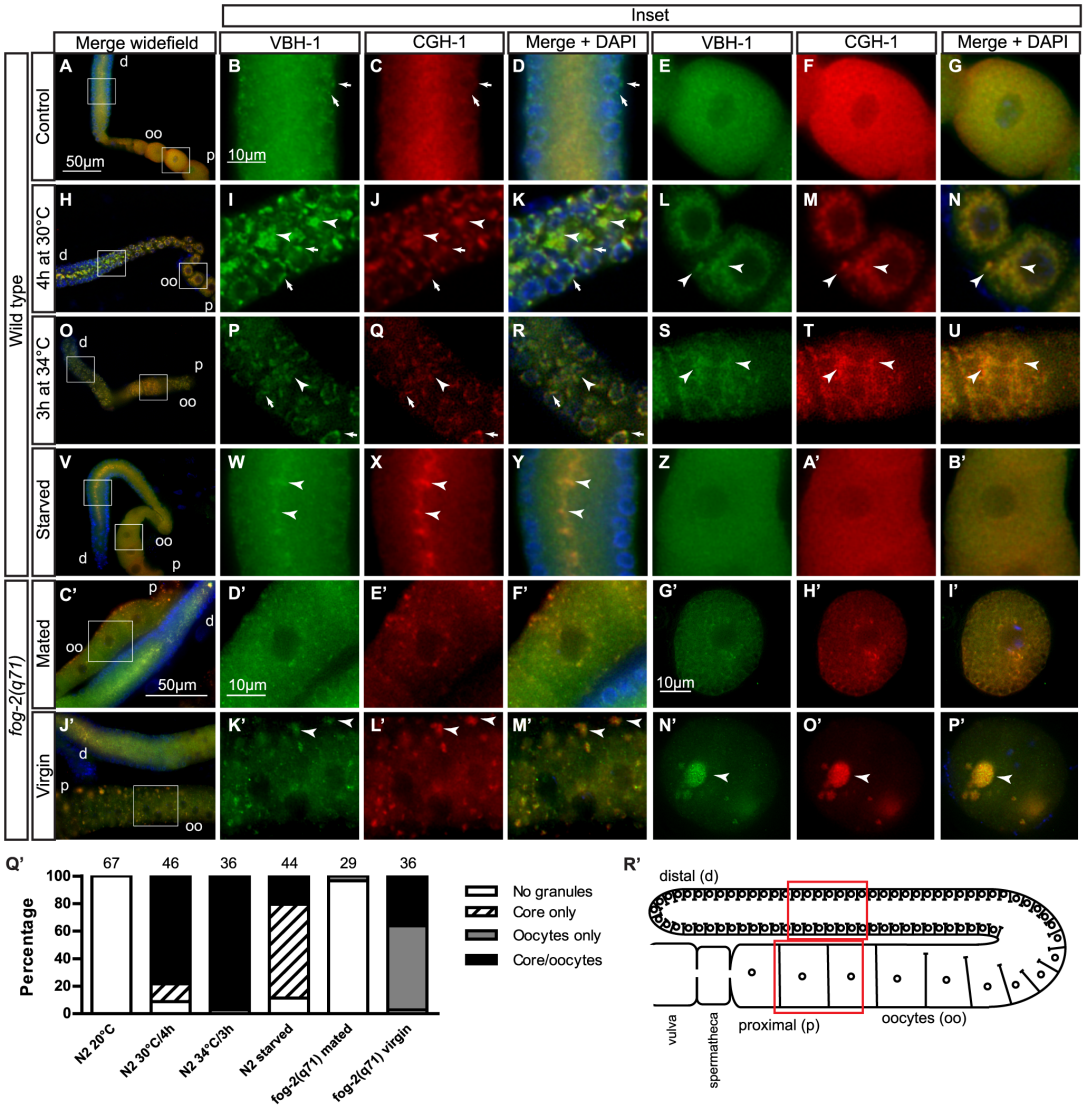
VBH-1 associates with CGH-1 into large foci in the gonad after stress. The extruded gonads of wild type and *fog-2(q71)* hermaphrodites were fixed and immunostained using anti-VBH-1 and anti-CGH-1 antibodies and DAPI to counterstain the DNA. Gonad of (A–G) N2 grown at 20°C, (H–N) N2 heat shocked for 4 h at 30°C, (O–U) N2 heat shocked for 3 h at 34°C, (V–B’) N2 placed on NGM plates without bacteria for 5 h, (C’–F’) feminized *fog-2(q71)* hermaphrodite mated, and (J’–M’) *fog-2(q71)* virgin animals. Oocytes from (G’–I’) *fog-2(q71)* hermaphrodite mated and (N’–P’) *fog-2(q71)* virgin animals. d =  distal, p =  proximal, oo =  oocytes, the arrows indicate P granules, and the arrowheads indicate the formation of foci after each stress. Scale bar in A applies to all wide field images of wild type gonads (A, H, O, and V), scale bar in B applies to all insets of wild type gonads (B–G, I–N, P–U and W–B), scale bar in C’ applies also to J’. Scale bar in D’ is the same for D’–F’ and K’–M’, and scale bar in G’ is the same for H–I’ and N’–P’. (Q’) Grouped graph summarizing the phenotypes observed, and the numbers above each bar indicate the number of animals analyzed. (R’) Cartoon of *C. elegans* gonad, representing the different regions. Red squares indicate regions of interest shown on insets.

**Figure 5:**
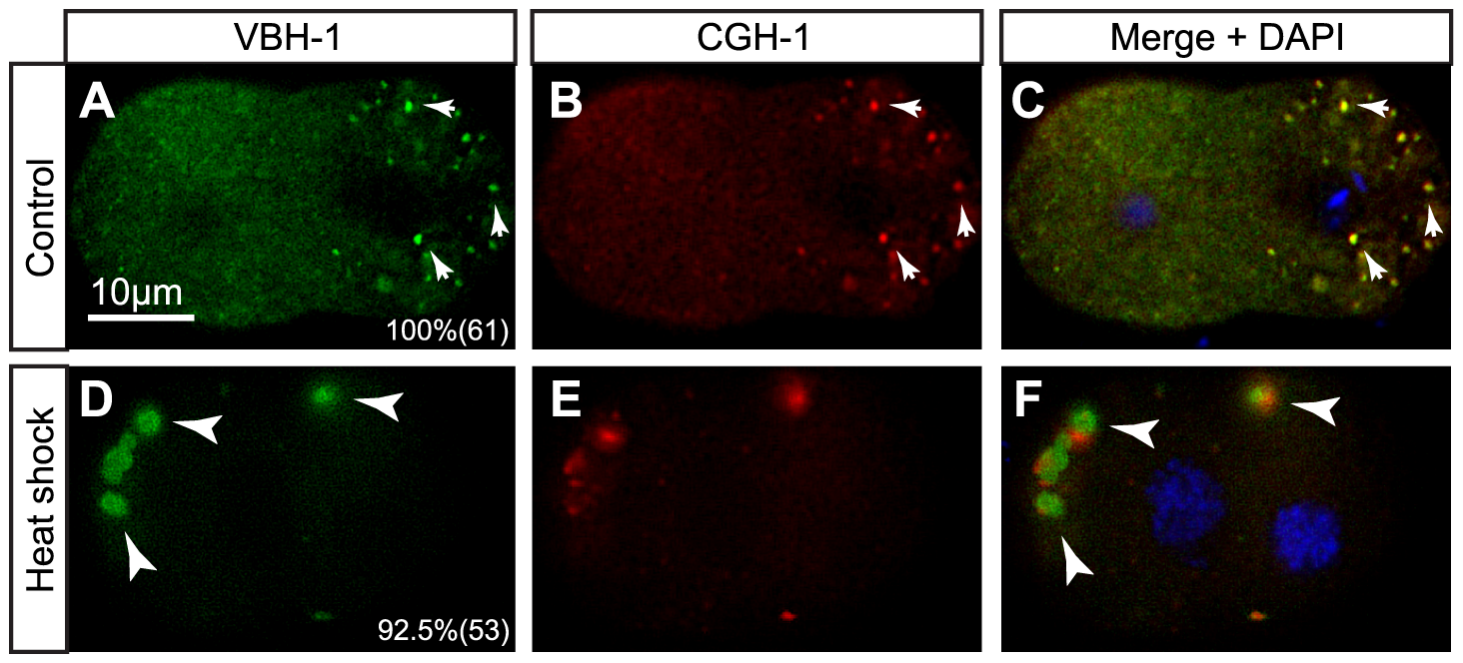
VBH-1 associates to large foci in early embryos after heat shock. The extruded embryos of wild type hermaphrodites were freeze cracked, fixed and immunostained using anti-VBH-1 and anti-CGH-1 antibodies and DAPI to counterstain the DNA. 2-cell embryos from N2 hermaphrodites (A–C) grown at 20°C and (D–F) heat shocked for 4 h at 30°C. The posterior pole of the embryo is oriented to the right. The arrows indicate P granules, the arrowheads indicate large foci, the numbers in the lower corner indicate the percentage where the depicted phenotype was observed and the number of embryos observed in parenthesis.

Extruded gonads and embryos from *xmSi05* adults (soma and germline expression) were placed on a slide and observed under an epifluorescence microscope to analyze the location of the transgene. We have previously reported that VBH-1 is expressed in cytoplasmic foci, perinuclear germ granules termed P granules in germ cells, and diffusely expressed throughout the cytoplasm of all blastomeres [3]. The GFP transgene showed the same expression as the endogenous VBH-1 in germline cells and embryos (compare Figure 3A and C to Figures 4B and E, and 5A) suggesting that the expression of the transgene was similar to endogenous VBH-1.

GFP::VBH-1 expression was observed in the intestine of *xmSi05* animals, but the high expression in the germline interfered with the observation. To overcome this complication, we used the *xmSi06* strain, which showed low expression in the germline. GFP::VBH-1 expression was observed in the cytoplasm of intestinal cells, with a clear enrichment in the apical pole in control animals (Figure 3F, arrows). The *C. elegans* intestine has autofluorescent lysosomes in the cytoplasm [38], which could be confused with GFP fluorescence. To overcome this effect, we silenced *vbh-1* with RNAi through feeding and compared the fluorescence in the intestines of RNAi animals to that in control animals. The fluorescent signal in the cytoplasm was reduced and the apical enrichment was not observed in *vbh-1(RNAi)* animals (Figure 3E), suggesting that the transgene is expressed in this region of the intestine. We next analyzed whether this localization changed during heat shock and observed that the cytosolic GFP signal in the intestinal cells was lower and became diffuse in the apical pole (Figure 3G, arrows). Although *xmSi06* animals show somatic *vbh-1* expression we cannot discard that this is an artifact of this transgene.

### The mRNAs of the heat shock proteins SIP-1 and HSP-1 are downregulated in *vbh-1(RNAi)* and *laf-1(RNAi)* animals

Given that VBH-1 is important during the stress response, we identified putative targets in a microarray analysis in which transcripts from one-day-old *vbh-1(RNAi)* adults were compared to those of control animals of the same age at 20°C (unpublished data). Two heat shock protein mRNAs, *sip-1* and *hsp-1*, were downregulated in this analysis. SIP-1 is a member of the alpha-crystallin/Hsp20 family, while HSP-1 is a member of the Hsp70 family [39], [40]. To confirm the abundance of *hsp-1* and *sip-1* mRNAs in control and *vbh-1(RNAi)* animals, we used semi-quantitative RT-PCR analysis. We observed that *sip-1* mRNA and, to a lesser extent, *hsp-1* mRNA were downregulated in *vbh-1(RNAi)* animals (Figure 2B).

Because LAF-1 was also important during stress, we decided to analyze whether these mRNAs were downregulated in *laf-1(RNAi)* animals. We observed a reduction in the abundance of *hsp-1* mRNA and, to a lesser extent, in *sip-1* mRNA in *laf-1(RNAi)* animals (Figure 2B). As expected, we also observed that *sip-1* and *hsp-1* mRNAs were downregulated in *vbh-1(RNAi); laf-1(RNAi)* animals (Figure 2B). We concluded that VBH-1 and LAF-1 regulate the accumulation of *sip-1* and *hsp-1* mRNAs. However, VBH-1 might be more important for the regulation of *sip-1* mRNA, while LAF-1 could be more important for the accumulation of *hsp-1* mRNA.

To determine whether HSP-1 and SIP-1 are important for heat shock survival, we used RNAi to silence the genes encoding these proteins and assessed the survival of these animals at 36°C. First, we confirmed the efficiency of *hsp-1* and *sip-1* silencing through semi-quantitative RT-PCR (Figure 2C). Similar to *vbh-1(RNAi)* animals, *sip-1(RNAi)* animals incubated at 36°C were more sensitive than the control animals (Figure 1B and Table S1). In contrast, we did not observed statistically significant differences in *hsp-1(RNAi)* animals (Figure 1B and Table S1). We conclude that SIP-1 is important for heat shock survival, while HSP-1 appears to be not essential. It is also possible that *hsp-1* is important for the heat shock response, but because we used RNAi silencing instead of a null mutant, we did not fully appreciate the role of this protein.

### VBH-1 associates with large granules during stress in *C. elegans* gonads and embryos

Because the expression of *vbh-1* is higher in the germline, we analyzed the subcellular localization of VBH-1 in the gonad during heat shock. To induce heat shock, one-day-old N2 adults were incubated for 4 h at 30°C. We used a milder heat shock condition instead of the conditions used in the survival assay (36°C) because the germline and embryos are sensitive to heat shock. The extruded gonads from animals subjected to heat shock were fixed, immunostained with an anti-VBH-1 antibody and observed under an epifluorescence microscope to analyze the location of VBH-1. The gonad and regions of interest shown as insets are indicated in Figure 4A, H, O, V, C’, J’, and R’.

At 20°C, VBH-1 was observed in P granules and cytoplasmic foci and was diffusely expressed throughout the cytoplasm of germ cells (Figure 4 A, B, and E, arrows) as previously reported [3]. After heat shock, VBH-1 was visible as aggregates in the gonad core and oocytes [Figure 4H, I, L (arrowheads) and Q], and in large P granules in the gonad [Figure 4H, I, K (arrows), and Q]. A similar aggregation pattern was also observed for GFP::VBH-1 during heat shock (Figure 3B arrowheads). Several types of RNP complexes have been described in the germline and embryos of control and stressed *C. elegans* [41]–[43]. These RNPs share some proteins, including the DEAD box RNA helicase CGH-1 [41]–[43]. To determine whether VBH-1 associates with these previously described RNPs during heat shock, the extruded gonads were also immunostained with an anti-CGH-1 antibody [44]. VBH-1 colocalized with CGH-1 in the gonad core granules and large P granules observed during heat shock (Figure 4H-N, arrowheads). These data suggest that VBH-1 and CGH-1 associate with some of the same RNPs during heat shock.

The formation of large RNP complexes in the gonad of *C. elegans* were previously observed using a heat shock temperature of 34°C [42] instead of 30°C; therefore we decided to analyze *vbh-1* expression at this temperature. We observed that VBH-1 also aggregated with CGH-1 in the gonad core and oocytes of animals incubated at 34°C for 3 h, but these aggregates were smaller and less evident that those formed at 30°C (Figure 4O–U). However, incubation at 34°C was more efficient in terms of percentage of animals that have aggregates in both the gonad core and oocytes (Figure 4Q’).

To determine whether VBH-1 and CGH-1 also aggregate in granules under other types of stress, we starved one-day-old hermaphrodites for 5 h and analyzed VBH-1 localization through immunofluorescent staining. In the gonads from starved animals, VBH-1 and CGH-1 were located in aggregates in the gonad core [Figure 4V–Y (arrowheads), and Q’], and these aggregates were smaller than those formed during heat shock (Figure 4H–K, arrowheads). Large P granules and oocyte aggregates were observed at a lower frequency under starvation (Figure 4V–B’, and Q’).

Ovulation arrest induces the formation of RNP granules in the *C. elegans* gonad [42], [45]. To determine whether VBH-1 changes this expression pattern under this condition, we used *fog-2(q71)* hermaphrodites. FOG-2 is an F-box-containing protein required in the hermaphrodite gonad to direct spermatogenesis, and *fog-2(q71)* hermaphrodites do not make sperm and its ovulation is arrested until mating [46], [47]. We immunostained the extruded gonads obtained from two-day-old mated and unmated *fog-2(q71)* adult hermaphrodites using anti-VBH-1 and anti-CGH-1 antibodies. VBH-1 colocalized with CGH-1 in large foci in the oocytes of virgin animals [Figure 4J’–P’ (arrowheads), and Q’), and these foci were not observed in mated *fog-2(q71)* hermaphrodites (Figure 4C’–I’). Aggregates were observed in the distal core of the gonad, but these clusters appeared at a lower frequency compared with those formed during heat shock (Figure 4Q’). Together, these results show that VBH-1 associates in foci with CGH-1 in the hermaphrodite gonad after exposure to different types of stress.

VBH-1 is also diffusely expressed during embryogenesis in the cytoplasm of all blastomeres and P granules in germline blastomeres throughout embryogenesis [3]. To characterize the differences in the embryonic localization of VBH-1 we exposed one-day-old hermaphrodites to 30°C for 4 h, and immunostained these embryos with anti-VBH-1 and anti-CGH-1 antibodies. At 20°C, VBH-1 colocalized with CGH-1 in P granules in the germline blastomeres of early embryos (Figure 5A–C, arrows) and was diffusely expressed throughout the cytoplasm of all blastomeres (Figure 5A–C) as previously described [3]. Following heat shock, VBH-1 associated with large granules in both somatic and germline blastomeres (Figure 5D, arrowheads). Interestingly, the localization of CGH-1 did not precisely overlap with VBH-1, but rather CGH-1 foci were close to VBH-1 aggregates (Figure 5D–F, arrowheads). These observations show that VBH-1 associates with large aggregates after fertilization that did not colocalize with CGH-1 foci. However, both foci remained closely associated, suggesting a potential interaction between them. GFP::VBH-1 showed localization patterns similar to endogenous VBH-1 in control and heat shocked embryos (Figure 3C and D), suggesting that the transgene reproduces the endogenous expression of *vbh-1*.

### Localization of VBH-1 to P granules depends on its helicase and C-terminus domains

**Figure 6:**
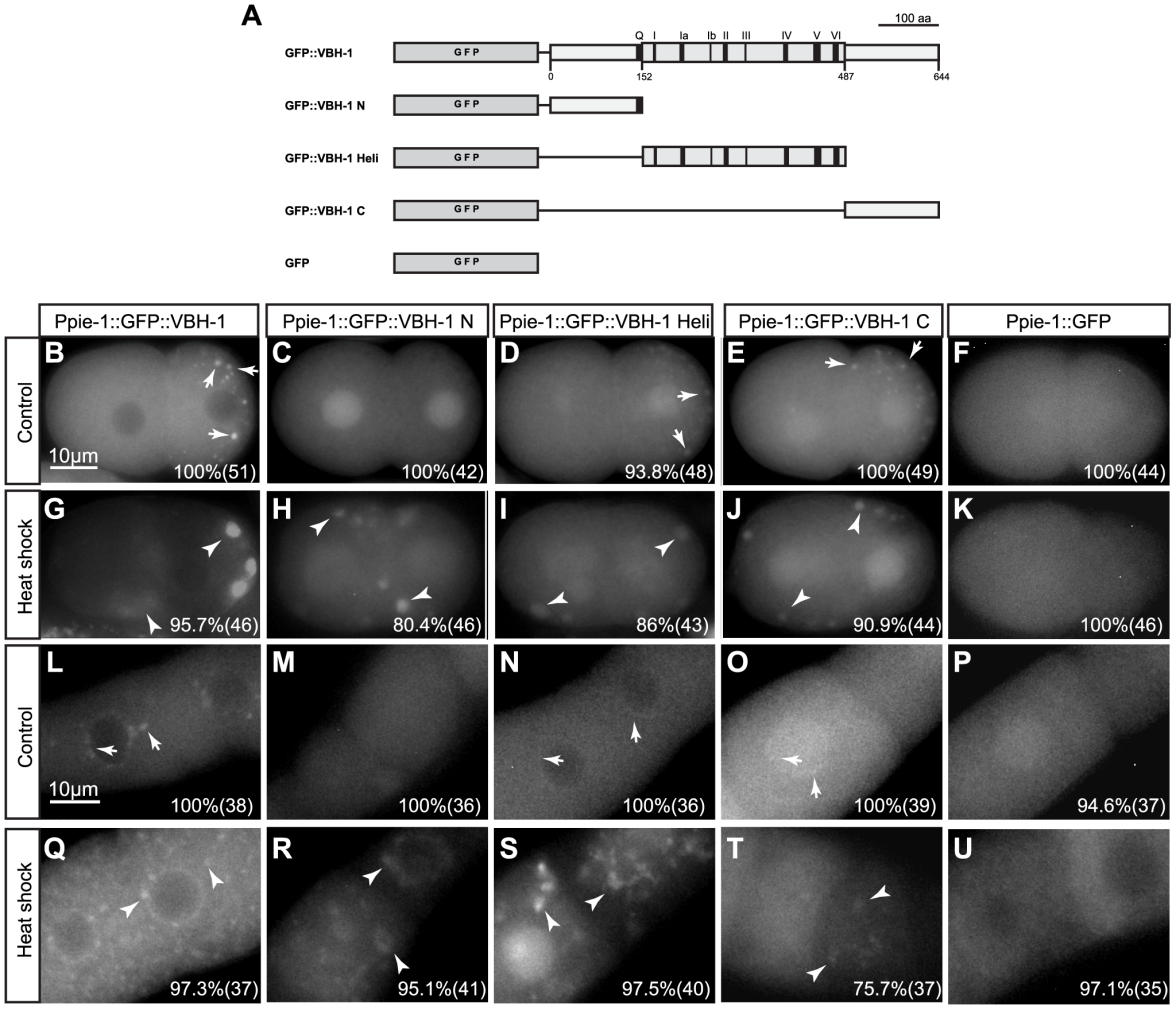
VBH-1 association with P granules depends on VBH-1 helicase and C-terminus domains. (A) Diagram of the transgenes constructed. Embryos from hermaphrodites expressing the indicated transgenes were mounted and microscopically observed under an epifluorescence microscope. (B–K) 2-cell embryos from (B–F) control and (G–K) heat shocked animals. (L–U) Oocytes from (L–P) control and (Q–U) heat shocked animals. The posterior pole of the embryo is oriented to the right. The arrows indicate P granules, the arrowheads indicate slarge foci, the numbers in the lower corner indicate the percentage where the depicted phenotype was observed and the number of samples observed is indicated in parentheses.

To determine the domain requirement of VBH-1 for association with foci, we fused different regions of VBH-1 to green fluorescent protein (GFP) and analyzed the localization of this protein. To generate these transgenes, we cloned the N-terminus (VBH-1 N), helicase domain (VBH-1 Heli) and C-terminus (VBH-1 C) of VBH-1 (Figure 6A) into the pID3.01B destination vector [3] containing the promoter and 3′UTR of *pie-1* to drive germline expression and the GFP coding gene at the N-terminus. Constructs were introduced to *C. elegans* by standard biolistic transformation [48]. The transgenic animals were grown at 24°C to avoid germline silencing [49], followed by dissection and subsequent observation under an epifluorescence microscope. Because we used the regulatory regions of *pie-1*, these transgenes showed low expression in the distal region of the gonad, and only the oocytes and embryos were analyzed. Under control conditions, GFP::VBH-1 Heli and GFP::VBH-1 C were localized to the cytoplasm and P granules in embryos and oocytes (Figure 6D–E and N–O, arrows). This localization resembles that of endogenous VBH-1 (Figure 4E and 5A) and the transgene containing the full VBH-1 ORF (Figure 6B and L, arrows) [3]. Although the GFP::VBH-1 Heli P granules formed in oocytes were consistently less evident than GFP::VBH-1 C and full length GFP::VBH-1 P granules (Figure 6L, N–O). In contrast, GFP::VBH-1 N was diffusely expressed in the cytoplasm without association with P granules (Figure 6C and M). These observations show that under normal growth conditions, the C-terminus and, to a lesser extent, the helicase domain of VBH-1 are sufficient for VBH-1 localization into P granules, while the N-terminus was dispensable for this localization.

We next analyzed the localization of the transgenes in oocytes and embryos from animals subjected to heat shock at 30°C for 4 h. The three GFP-fused fragments of VBH-1 associated into large aggregates in somatic and germline blastomeres (Figure 6H–J, arrowheads) and oocytes (Figure 6R–T, arrowheads), as observed for the full VBH–1 transgene (Figure 6G and Q, arrowheads). This could suggest that the aggregation of VBH-1 under this condition does not depend on a specific domain but might reflect multiple interactions with other molecules. We also observed that, during heat shock, the GFP::VBH-1 C foci in oocytes were less visible and frequent that those formed by the other two fragments tested (Figure 6R–T). We conclude that during heat shock, the N-terminus and helicase domains of VBH-1 seem to be sufficient for the localization of VBH-1 into granules in both oocytes and embryos, while the C-terminus seems to be less important for the association of VBH-1 into granules in oocytes than in early embryos.

To discard a GFP artifact in granule formation, we made a transgene that did not carry VBH-1 domains (*Ppie-1::GFP::pie-1 3*′*utr*) as a negative control. We did not observe the association of this transgene with P granules under control temperatures or the aggregation of this protein with large foci during stress (Figure 6F, K, P and U). These results suggest that foci or aggregations were not induced through GFP but are the results of VBH-1 properties. We observed a different cytoplasmic distribution of GFP (Figure 6U) in the oocytes of animals subjected to heat shock, which does not correspond to VBH-1 aggregates.

We observed nuclear localization of some of the transgenes carrying different VBH-1 fragments and, to a lesser extent, of the GFP transgene alone (Figure 6). Neither the endogenous VBH-1 nor the full VBH-1 transgene were localized in the nucleus (Figure 4, 5, 6B, G, L, and Q); therefore we think that the nuclear localization of the transgenes could be due to non-specific localization.

## Discussion

In the present study, we analyzed the role of the DEAD box protein VBH-1 during stress. We observed that VBH-1 is important for the survival of *C. elegans* during heat shock and oxidative stress. Our results suggest a potential somatic expression of *vbh-1* that could be at least partially responsible for stress survival. We found that *sip-1* and *hsp-1* mRNAs, which encode two heat shock proteins, were downregulated in *vbh-1(RNAi)* animals, suggesting that these proteins could either be directly or indirectly regulated through VBH-1. Furthermore, we observed that LAF-1 (a close homolog of VBH-1) is also important for survival during stress, and *hsp-1* and *sip-1* mRNA were also downregulated in *laf-1(RNAi)* animals. In addition, we observed that VBH-1 aggregates in early embryos, the gonad core, oocytes and large P granules in heat-shocked animals. We also observed that the core helicase and C-terminal domains of VBH-1 are sufficient for association with P granules, while the aggregation of VBH-1 during stress does not depend on a specific motif. Although the N-terminus and helicase domains seem to be more important for this localization.

### VBH-1 protects *C. elegans* from stress through the regulation of the stress response mRNAs *sip-1* and *hsp-1*

In the present study, we have shown that *vbh-1* silencing had a negative effect on the survival of animals during heat and oxidative stresses (Figure 1B–D). We suggest that the mRNAs of the heat shock proteins SIP-1 and HSP-1 are candidates of VBH-1 regulation during the stress response because 1) *sip-1* mRNA and, to a lesser extent, *hsp-1* mRNA were downregulated when *vbh-1* was silenced using RNAi, and 2) *sip-1* silencing negatively affected the survival of heat shocked animals in a manner similar to that of *vbh-1(RNAi)* animals. *hsp-1(RNAi)* animals were not more sensitive to heat shock than control animals, suggesting that *hsp-1* is not required for a proper response to this stress, but it is also likely that *hsp-1(RNAi)* is not sufficient to completely eradicate the function of this protein during stress.

In other organisms, VBH-1 homologs are involved in the regulation of genetic expression through mRNA stabilization and translation [29], [50]; therefore, it is possible that VBH-1 could be required for the stability or translation of *sip-1* and *hsp-1* mRNAs. It is likely that *sip-1* mRNA might require a protein, such as VBH-1, for protection from degradation as the transcription of this gene does not increase during heat shock [39], unlike other heat shock protein-coding genes, such as *hsp-1* [40].

VBH-1 could also regulate the translation of *sip-1* and *hsp-1* mRNAs. In *D. melanogaster*, the VBH-1 homolog Vasa positively regulates the translation of RNAs that have a polyU tract in the 3′UTR. Interestingly, the mRNA of *hsp-1* has a polyU tract in the 3′UTR (Figure S1), suggesting VBH-1 might regulate the translation of this protein if a similar regulation mechanism is conserved in VBH-1. In addition, the human VBH-1-related protein, DDX3, promotes the translation of stress related mRNAs possessing internal ribosome entry sites (IRES) [51]. The 5′UTR of *sip-1* mRNA does not have predicted IRES sites [52], while the 5′UTR of *hsp-1* is only 5 bp (http://www.wormbase.org, release WS236, May 8, 2013); therefore, it is unlikely that VBH-1 regulates the translation of these mRNAs through this mechanism. However, *hsp-3* [53] in *C. elegans* and Hsp70 proteins in *D. melanogaster* [54] and human [55] have IRES.

A third possibility is that VBH-1 might indirectly regulate the stability of *sip-1* and *hsp-1* mRNAs through the regulation of another gene, although the regulation of heat shock proteins through VBH-1-related helicases is conserved in other organisms. For example, in *D. melanogaster,* Vasa co-immunoprecipitates with the mRNAs of eight chaperones, two of which are members of the same families as SIP-1 and HSP-1, i.e., alpha-crystallin and Hsp70 [16]. Another example is the only DEAD box RNA helicase encoded in the cyanobacteria *Synechocystis* genome, CrhR, which regulates the stability of the chaperonines *groES*, *groEL1*, and *groEL2* mRNAs required for a proper response to stress [56].

The VBH-1 paralog LAF-1 is important for survival during stress and is also important for the accumulation of *hsp-1* and, to a lesser extent, *sip-1* transcripts. However, we did not observed an additive effect in survival when we silenced both genes, suggesting that the roles of VBH-1 and LAF-1 in stress responses might not be redundant. Given that RNAi-mediated silencing is not as efficient as a mutation, it is possible that both RNA helicases could be redundant during the stress response.

VBH-1 is also important in the induction of apoptosis in the germ cells under both normal and stress conditions [5], suggesting that this protein could have several targets during the stress response in *C. elegans* to ensure the survival of the animals, maintain oocyte quality, and preserve germline cells.

### The potential somatic expression of *vbh-1* could protect *C. elegans* from stress

Using the germline-depleted mutant *glp-4(bn2)*, we observed that *glp-4(bn2)* animals are more resistant to heat shock than wild type animals. Accordingly, it has been reported that *glp-4(bn2)* animals show an enhanced survival when exposed to anoxia or Gram-negative bacterial pathogens [57], [58]. These results suggest that germline-deficient mutants might have increased stress resistance.

We also found that the somatic expression of *vbh-1* was important for the survival of *C. elegans* under stress. We could not rule out that the expression of *vbh-1* in the germline could also play a role in stress survival given that 1) the difference between control and *vbh-1(RNAi)* animals is smaller in *glp-4(bn2)* than in wild type animals, 2) *glp-4(bn2*) grown at a restrictive temperature retains approximately 12 germ nuclei [34], and 3) the germline can signal to the soma and control lifespan [57], [59], [60].

This finding was unexpected because we have previously reported that VBH-1 was not detected in *glp-4(bn2)* animals based on Western blotting [3]. Although in this mutant, we detect a small expression of *vbh-1* mRNA through Northern blotting [3]. It is possible that the low somatic expression of *vbh-1* could not be detected through Western blotting or that our VBH-1 antibody does not detect somatic VBH-1 due to possible posttranslational modifications. We observed that GFP::VBH-1 is localized in the intestine of *C. elegans*, in which stress response proteins are commonly expressed [61]. Furthermore, the transgene is expressed in the apical pole of the intestinal cells, which express HSP16 family chaperones belonging to the same group as SIP-1 under stress [62]. However, we cannot rule out that this localization is just an artifact of the transgene. Following stress, GFP::VBH-1 was enriched in the apical pole of intestinal cells, but this expression was more diffusely distributed. It is possible that this difference in the localization might be associated with the protective role of VBH-1 during stress.

### VBH-1 associates with CGH-1 foci in the gonads and embryos upon stress

Under normal growth conditions, VBH-1 is diffusely expressed in the germ cell cytoplasm and associates with P granules [3]. Here, we showed that following stress, VBH-1 aggregates into foci in the gonad core and oocytes, where it associates with CGH-1. CGH-1 associates with several RNPs formed in the *C. elegans* gonad during stress [41]–[43], [63] and the fact that VBH-1 colocalizes with CGH-1 suggests that these proteins might associate with the same RNPs during stress. Similar results have been observed with human DDX3, which associates with RNP complexes when overexpressed or after stress [64]–[66].

VBH-1 was also localized into large aggregates in both somatic and germline blastomeres during early embryogenesis after heat shock. The aggregates in somatic blastomeres are not P granules because these are asymmetrically inherited to the germline blastomeres and further disassembled and degraded through autophagy in somatic blastomeres [67], [68]. One important difference between the aggregates formed in the gonad and those observed in early embryos is that in the latter, VBH-1 did not perfectly overlap with CGH-1; however, these proteins are closely associated. Depending on its interacting partners, CGH-1 could stabilize mRNA and repress translation or play a role in mRNA decay [63]. This suggests that during stress VBH-1 and CGH-1 could share a role in the germline, while after fertilization these proteins could play separate roles.

We observed that both the conserved helicase core and C-terminal domains of VBH-1 associate with P granules in oocytes and early embryos, suggesting that both regions are responsible to drive VBH-1 into these structures. This observation suggests that the localization of VBH-1 depends on binding to RNA, as both regions contain RNA binding motifs: the C terminal domain has three Glycine-rich RGG motifs [3], [69], while the helicase region has motifs Ia, Ib, IV and V [50]. Unexpectedly, three different non-overlapping VBH-1 fragments associated into large foci during heat shock in oocytes and early embryos, suggesting that this association does not depend on a single interaction. However, the C-terminus of VBH-1 had less efficiency of foci formation in oocytes suggesting that the N-terminus and helicase domains are more important for the aggregation of VBH-1 during stress.

The three VBH-1 fragments tested were visible inside the nuclei of all blastomeres; however, the full *vbh-1* transgene expressed under the same promoter did not have nuclear localization. The GFP transgene was observed in the nuclei and cytoplasm of early embryos, but this protein was not as enriched in the nuclei as the other transgenes carrying VBH-1 fragments. In addition, immunostaining did not reveal endogenous VBH-1 in the nucleus, and VBH-1 does not have a predicted nuclear localization signal, unlike other members of the Belle/DDX3 family [8]. These findings suggest that nuclear localization of the transgenes could be due to non-specific localization.

## Materials and Methods

### *C. elegans* strains and growth conditions

*C. elegans* strains were maintained according to standard procedures [70]. The following *C. elegans* strains were used: the wild type variety Bristol strain N2, *fog-2(q71)* [46], *glp-4(bn2)* [34], *unc-119(ed3)* [71], EG6699 *[ttTi5605 II; unc-119(ed3)* III; *oxEx1578]* [36], RN007 *xmSi05 [Pvbh-1::GFP::vbh-1::vbh-1 3*′*utr, Cb unc-119(+)] II* (this study), and RN008 *xmSi06 [Pvbh-1::GFP::vbh-1::vbh-1 3*′*utr, Cb unc-119(+)] II* (this study), *Ppie-1::GFP::vbh-1::pie-1 3*′*utr* [3]. *Ppie-1::GFP::vbh-1 N::pie-1 3*′*utr* (this study), *Ppie-1::GFP::vbh-1 heli::pie-1 3*′*utr* (this study), *Ppie-1::GFP::vbh-1 C::pie-1 3*′*utr* (this study), and RN013 *xmSi24 [Ppie-1::GFP::pie-1 3*′*utr]* (this study).

### Cloning

Fragments corresponding to nucleotides 1610 through 1895 in the open reading frame (ORF) from the isoform a of *vbh-1* and nucleotides 125 through 308 in the ORF from the isoform a of *laf-1* (http://www.wormbase.org, release WS236, May 8, 2013) were PCR amplified using primers with restriction sites added to the 5′ end. The following primers were used, and the restriction sites sequences are indicated in lowercase: for *vbh-1* 5′-gaagatcttcGTCGTGGTGGTGGTGGATCG-3′ and 5′-gctctagagcGGAGCCTGGCCTGGGTTTG-3′; and for *laf-1* 5′-gaattccGTGGAAACCGTGGATACAATAATAATCG-3′ and 5′-ggggtaccccCCATCGCCTCCATTATCCCG-3′. The purified PCR products were digested with BglII and XbaI for *vbh-1* and EcoRI and KpnI for *laf-1*. The digested fragments were cloned into the pPD129.36 vector [72] previously digested with the same restriction enzymes. For the double *vbh-1* and *laf-1* RNAi, we digested the pPD129.36*(vbh-1)* plasmid with PvuII and PciI to obtain the *vbh-1* fragment together with the flanking T7 promoters and subcloned this fragment into the region of the pPD129.36*(laf-1)* vector between the TfiI (blunt-ended) and PciI restriction sites. All plasmids were confirmed through sequencing.

### RNA interference

RNAi silencing was performed through feeding at 25°C using standard methods [32], [73]. Briefly, RNAi vectors were transformed into the *E. coli* strain HT115(DE3). The transformed bacteria were cultured overnight in LB broth containing 50 µg/ml ampicillin and 12.5 µg/ml tetracycline. The dsRNA synthesis was induced overnight on NGM plates supplemented with ampicillin (50 µg/ml) and IPTG (1 mM). Subsequently, gravid N2 Bristol or *glp-4(bn2)* animals were collected in M9 buffer and lysed with a mixture of 5 N NaOH and bleach (1:2) using standard procedures [74]. The obtained embryos were hatched overnight in M9. L1 larvae were placed onto NGM plates containing induced bacteria and incubated at 25°C until adulthood. To induce RNAi in *sip-1* and *hsp-1*, the clones were obtained from the RNAi library (Open Biosystems) and confirmed through sequencing [75].

### Survival assays

Heat shock was performed as previously described [33], with some modifications. Briefly, synchronized L1 worms were incubated at 25°C on NGM plates prepared for RNAi for 40 h and subsequently transferred to several 35 mm NGM plates for heat shock (30–40 worms per plate). The plates were incubated for up to 10 h at 36°C, and every hour a plate was taken from the incubator to observe animals under a stereoscopic microscope. Animals without pharyngeal pumping that did not respond to touch were scored as dead, while burst animals were discarded. The data from different experiments were obtained, and the percentage of the total was graphed. Eight experiments were conducted for EP and *hsp-1(RNAi)*, six for *laf-1 (RNAi),* five for *vbh-1(RNAi),* four for *vbh-1(RNAi); laf-1(RNAi)*, and three experiments were conducted for *sip-1(RNAi)* and *glp-4(bn2)* animals.

To induce oxidative stress, we placed 30–40 RNAi animals onto 35 mm NGM plates layered on top with 20 µl of 15% hydrogen peroxide, and the dead animals were scored after 20 min at 25°C. The average from three different experiments was graphed. Statistical analysis and graphing were conducted using Prism software (GraphPad).

### RT-PCR

The RNA was purified using TRIzol (Life Technologies) and used as a template to synthesize cDNA using the ImProm-II Reverse Transcriptase kit (Promega) according to the manufacturer's instructions. The obtained cDNA was used in a PCR reaction using the following primers: for *hsp-1* 5′-CATGGTCAACGAAGCTGAGA-3′ and 5′-TTCCAAATCCTTCTGTTGGTG-3′; for *sip-1* 5′-ACAACATCGTGCCACAACAG-3′ and 5′-TGGTCATCTGTCCTTCCTTG-3′; for *vbh-1* 5′-CGCCAACGGAACTCCGTCAAAC-3′ and 5′-CCGGCCCAAAACCAGCTTCATT-3′, and for *laf-1* 5′-GTCGCGATCACCGTTATCAA-3′ and 5′-CTCCACCAATCCTGTTGAGG-3′.

### Immunostaining

Immunostaining was performed as previously described [44]. Briefly, extruded gonads and embryos were freeze-cracked and fixed with ice-cold methanol for 1 min, followed by treatment with 1X PBS, 3.7% formaldehyde, 80 mM HEPES, 1.6 mM MgSO_4_ and 0.8 mM EGTA at room temperature for 20 min. The samples were washed in PBT (PBS, 0.5% Tween 20) and blocked for 30 min in 30% normal goat serum with 0.2% azide in PBT. The samples were incubated with the indicated primary antibody overnight at 4°C, followed by 1 h of incubation at room temperature with the secondary antibody. The following antibodies and dilutions were used: rabbit anti-VBH-1 (1:100) [3], rat anti-CGH-1 (1:30) [44], FITC conjugated goat anti-rabbit IgG (Jackson Immunoresearch, 1:100) and Cy3 conjugated goat anti-rat IgG (Jackson Immunoresearch, 1:100). The DNA was counterstained with DAPI (4′, 6-diamidino-2-phenylindole, 1 ng/µl) and mounted with Vectashield Mounting Medium (Vector Labs) to prevent photobleaching.

### Image acquisition and processing

The images were obtained on a Nikon Eclipse E600 microscope equipped with an AxioCam MRc camera (Zeiss). The pictures were captured using AxioVision software (Zeiss) and deconvolved using ImageJ software [76] with Parallel Spectral Deconvolution and Diffraction PSF 3D plugins. The deconvolved images were processed using ImageJ and Illustrator CS5 software (Adobe).

### Transgene construction

Cloning *vbh-1* for biolistic transformation was performed using Gateway technology (Invitrogen). The cDNA clone yk624f9, obtained from the Kohara Lab, was used as a template for PCR as previously described [3]. The following primers were used: 5′-GGGGACAAGTTTGTACAAAAAAAGCAGGCTAACACACAATATTATGCG-3′ and 5′-GGGGACCACTTTGTACAAGAAAGCTGGGTTTTCTGGACAGGAGTCGG-3′ for *vbh-1 N*, 5′-GGGGACAAGTTTGTACAAAAAAAGCAGGCTCCGACTCCTGTCCAGAAAC-3′ and 5′-GGGGACCACTTTGTACAAGAAAGCTGGGTGATTCCTCGATTCTTGTC-3′ for *vbh-1 heli*, and 5′-GGGGACAAGTTTGTACAAAAAAAGCAGGCTGACAAGAATCGAGGAATC-3′ and 5′-GGGGACCACTTTGTACAAGAAAGCTGGGTTTAAGCTTGTGGAGCTTG-3′ for *vbh-1 C*. The PCR products were purified and cloned into the donor vector pDONR201. The fragments cloned were further recombined into the expression vector pID3.01b obtained from the Seydoux lab. The obtained plasmids were confirmed through sequencing.

The cloning of *vbh-1* for single-copy insertion was performed using the Multisite Gateway Three Fragment Vector Construction Kit (Invitrogen). For the promoter, we amplified a 608 bp fragment of the intergenic region upstream *vbh-1*, and for the coding region containing the 3′UTR, we amplified the region from the ATG to the end of the longest 3′UTR reported (http://www.wormbase.org, release WS236, May 8, 2013). The following primers were used: 5′-GGGGACAACTTTGTATAGAAAAGTTGCAGCTTTTGTGGTGCGTGACC-3′ and 5′-GGGGACTGCTTTTTTGTACAAACTTGTCATTGTAAGACTATATCAAACGG-3′ for promoter; and 5′-GGGGACAGCTTTCTTGTACAAAGTGGCAATGAACACACAATATTATGCGAATCACAACGG-3′ and 5′-GGGGACAACTTTGTATAATAAAGTTGGACTATTTGTCAGGCCACAAGGGC-3′ for the coding region. The PCR products were purified, and the promoter fragment was cloned through recombination in the donor vector pDONRP4-P1R, while the coding region was cloned into pDONRP2R-P3 to generate entry clones. In both cases, genomic DNA was used as a template, and the inserts were confirmed through sequencing. For the GFP sequence, we used the entry clone pCM1.53 obtained from the Seydoux Lab 2007 vector kit (Addgene plasmid 17250) [77].

For the *Ppie::GFP::pie-1 3*′*utr* transgene we used the following vectors from the Seydoux lab: pCM1.127 (pie-1 promoter, Addgene plasmid 21384), pCM1.53 (GFP, Addgene plasmid 17250), and pCM5.47 (pie-1 3′utr, Addgene 17254). The three fragments were cloned in tandem through recombination into the pCFJ150 vector obtained from the Jorgensen lab (Addgene plasmid 19329) [36]. The plasmid were confirmed through sequencing.

### Generation of transgenic strains

The fragments cloned into the pID3.01b vector were bombarded into *unc-119(ed3)* animals using standard procedures [48]. The BioRad Biolistic PDS-1000/He particle delivery system with Hepta adapter was used at 1500 psi. Animals that lost the Unc-119 phenotype and expressed GFP were selected.

*Pvbh-1::GFP::vbh-1::vbh-1 3*′*utr* transgenic animals were generated through Mos1-mediated single copy insertion [35], [36]. Briefly, one-day-old EG6699 (*ttTi5605*) adults were microinjected using a mixture containing the following plasmids: pCFJ150 with the transgene *Pvbh-1::GFP::vbh-1::vbh-1 3′utr* cloned (10 ng/µl), pCFJ601 (*Peft::Mos1 transposase*, 10 ng/µl), the negative selection marker pMA122 (*Phsp-16.1::peel-1*, 10 ng/µl), and the co-injection markers pCFJ104 (*Pmyo-3::mCherry*, 10 ng/µl), pGH8 (*Prab-3::mCherry*, 10 ng/µl), and pCFJ90 (*Pmyo-2::mCherry*, 2 ng/µl). The injected worms were incubated at 24°C for several days, and the *unc-119(+)* animals were heat shocked at 34°C for 2 h. The living animals were isolated, and their progeny was analyzed using a fluorescence microscope. GFP-expressing animals with no mCherry expression were selected.

*Ppie-1::GFP::pie-1 3*′*utr* transgenic animals were generated in a similar manner using the following modifications in the injection mix: pCFJ150 (20 ng/µl), pCFJ601 (50 ng/µl), pMA122 (10 ng/µl), pCFJ104 (5 ng/µl), pGH8 (10 ng/µl), and pCFJ90 (2.5 ng/µl).

## Supporting Information

Figure S1 The 3′UTR of *hsp-1* mRNA has polyU tracts. 3′ UTR of *hsp-1* mRNA, and the Uridines are highlighted in gray for clarity.
(TIF)

Table S1 Summary of survival data.
(DOC)
