# Supplementary figures and images for: The DEAD Box RNA Helicase VBH-1 Is a New Player in the Stress Response in C. elegans
Source: PLoS One. 2014 May 20;9(5):e97924. doi: 10.1371/journal.pone.0097924 (PMC4028217; doi:10.1371/journal.pone.0097924)

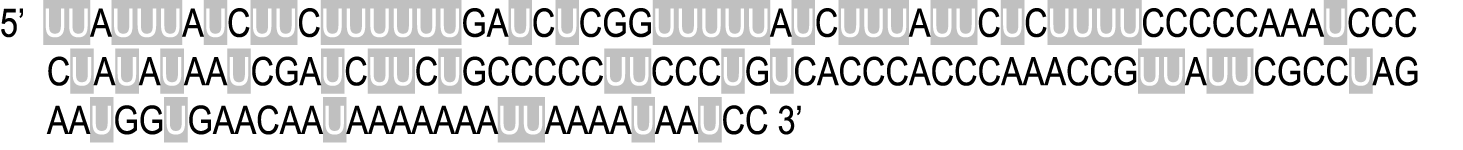

Supplement: Figure S1 — The 3′UTR of hsp-1 mRNA has polyU tracts. 3′ UTR of hsp-1 mRNA, and the Uridines are highlighted in gray for clarity. (TIF) [file pone.0097924.s001.tif]
