# Supplementary material for: The DEAD Box RNA Helicase VBH-1 Is a New Player in the Stress Response in C. elegans
Source: PLoS One. 2014 May 20;9(5):e97924. doi: 10.1371/journal.pone.0097924 (PMC4028217; doi:10.1371/journal.pone.0097924)
Supplement: Table S1 — Summary of survival data. (DOC) [file pone.0097924.s002.doc]

**Table S1. Summary of survival data.**

|  | **Time (h)** | | | | | | | | |
| --- | --- | --- | --- | --- | --- | --- | --- | --- | --- |
| **Background** | 2 | 3 | 4 | 5 | 6 | 7 | 8 | 9 | 10 |
| **N2, EP** | 100%  (287) | 100%  (280) | 96.6%  (294) | 88.8%  (295) | 76.9%  (282) | 54.7%  (212) | N.D. | N.D. | N.D. |
| **N2, *vbh-1(RNAi)*** | 100%  (165) | 93.6%  (159) | 71.2%  (163)*** | 62.1%  (161)*** | 43.9%  (164)*** | 5%  (120)*** | N.D. | N.D. | N.D. |
| **N2, *laf-1(RNAi)*** | 100%  (211) | 100%  (206) | 89.9%  (209) | 80.5%  (210) | 54.6%  (185)*** | 36.5%  (192)** | N.D. | N.D. | N.D. |
| **N2, *vbh-1(RNAi); laf-1(RNAi)*** | 100%  (120) | 91.6%  (119) | 71.7%  (120)*** | 62.1%  (116)*** | 40.5%  (116)*** | 16.7%  (84)*** | N.D. | N.D. | N.D. |
| **N2, *sip-1(RNAi)*** | 100%  (98) | 91.9%  (99) | 72.2%  (97)*** | 70.3%  (111)* | 46.8%  (94)*** | 23.2%  (95)*** | N.D. | N.D. | N.D. |
| **N2, *hsp-1(RNAi)*** | 100%  (284) | 99.6%  (285) | 89.7%  (291) | 81.7%  (268) | 77.3%  (278) | 58%  (248) | N.D. | N.D. | N.D. |
| ***glp-4(bn2)*, EP** | 100%  (84) | 100%  (91) | 98.7%  (149) | 94%  (151) | 89%  (191) | 82.8%  (192) | 74.7%  (87) | 64.4%  (101) | 60.9%  (110) |
| ***glp-4(bn2); vbh-1(RNAi)*** | 100%  (87) | 98%  (101) | 94.2%  (138) | 83.3%  (132)* | 69.3%  (192)*** | 57.6%  (184)*** | 49.5%  (97)*** | 30.7%  (101)*** | 18.8%  (64)*** |

Summary of the survival data at 36°C from Figures 1B and C. The numbers indicate the percentage of living animals and the number of animals analyzed in parentheses. Five experiments were conducted for EP, *vbh-1(RNAi)*, and *hsp-1(RNAi)*, four experiments were conducted for *vbh-1(RNAi); laf-1(RNAi)* and three experiments were conducted for *laf-1 (RNAi)* and *sip-1(RNAi)* and *glp-4(bn2).* Two-way ANOVA analysis with Bonferron’s test was used to compare each condition to the control. * P<0.05, ** P<0.01, *** P<0.001, N.D. not determined.
